# The Calibration of a Failure

**DOI:** 10.1007/s11606-026-10636-8

**Published:** 2026-07-29

**Authors:** Jason Miguel Bean

**Affiliations:** Baylor University, Waco, TX USA

The treadmill does not care about your morning. It is already moving, a black rubber tongue licking the soles of your Nikes, waiting for the gait to stutter.

In the lab, we wait for the blood to turn. That sharp, metallic bloom in the back of the throat, where breath becomes something the body has to fight for.

Yesterday: three hours calibrating the VO_2_ mask. A plastic muzzle, cold and smelling of isopropyl, strapped to a sophomore who still believes the body is negotiable.

He wants his peak.

I want the threshold.

The decimal place where muscle breaks free of the mind.

Green lines climb the screen, jagged and impatient. Outside, the Baylor sun washes Marrs McLean in gold. Inside, the room is salt, recycled air, and someone else’s effort clinging to the walls.

“Five more seconds,” I say.

The protocol needs ten.

Just enough time to watch the eyes lose focus, to see what stays when the will slips.

When he folds toward the rails, the data is clean.

A vertical drop.

I unstrap the mask and hand him a towel. He shakes, searching for breath like a lost key. The monitor drains to grey.

We call this failure: the cleanest point on the graph, the moment the body stops permitting pain.

## Data Availability

Data availability is not applicable to this article because no datasets were generated or analyzed.

